# Efficacy and safety of darbepoetin alfa initiated at hemoglobin ≤10 g/dL in patients with stage IV cancer and chemotherapy‐induced anemia

**DOI:** 10.1002/cam4.958

**Published:** 2016-11-23

**Authors:** Ralph V. Boccia, David H. Henry, Laura Belton, Chet Bohac, Hassan H. Ghazal

**Affiliations:** ^1^ Center for Cancer and Blood DisordersBethesdaMaryland; ^2^ University of PennsylvaniaPhiladelphiaPennsylvania; ^3^ LB BiostatisticsLondonUnited Kingdom; ^4^ Amgen Inc.Thousand OaksCalifornia; ^5^ Kentucky Cancer ClinicHazardKentucky

**Keywords:** Chemotherapy‐induced anemia, darbepoetin alfa, hemoglobin, meta‐analysis

## Abstract

Data on efficacy and safety of darbepoetin alfa (DA) administered at hemoglobin (Hb) ≤10 g/dL are limited. In this analysis, we examined DA's efficacy and safety in patients with Stage IV cancers and chemotherapy‐induced anemia (CIA) initiated on DA at Hb ≤10 g/dL. Data for patients with Stage IV cancers and CIA and who initiated DA at Hb ≤10 g/dL were extracted from three phase 3 trials identified in a central database of Amgen‐sponsored DA studies in CIA. Efficacy outcomes were assessed by achievement of Hb increases of ≥1 g/dL and ≥2 g/dL and red blood cell (RBC) or whole blood transfusion requirements. Data were analyzed for all patients with baseline Hb ≤10 g/dL, and by the subgroups of patients with baseline Hb ≥9 to ≤10 g/dL versus <9 g/dL. Crude and Kaplan–Meier proportions of patients who experienced each outcome and time (days) to each outcome were summarized by treatment. Meta‐analysis (fixed‐effects inverse‐variance model) was performed to compare outcomes for DA versus placebo. Safety was assessed by occurrence of adverse events. Data from 213 patients were analyzed: DA, *n *= 115; placebo, *n *= 98. More patients in the DA versus the placebo subgroup achieved Hb increase of ≥1 g/dL (72% vs. 36%; HR: 2.92, 95% CI: 1.95, 4.39) and ≥2 g/dL (44% vs. 18%; HR: 2.98, 95% CI: 1.71, 5.21) during the first 12 treatment weeks. Median times to Hb increase of ≥1 g/dL and ≥2 g/dL were 36 days and 78 days for DA, respectively. RBC or whole blood transfusions were less common in patients in the DA versus the placebo subgroup (24% vs. 45%; HR: 0.44, 95% CI: 0.27, 0.73). No new safety issues were reported. Our results confirm that DA use in patients with Stage IV cancer and CIA is more effective than placebo at increasing Hb levels and at reducing transfusion needs when DA treatment is initiated at Hb ≤10 g/dL.

## Introduction

Patients with cancer who are treated with myelosuppressive chemotherapy often develop chemotherapy‐induced anemia (CIA) 1, 2. CIA often leads to the need for blood transfusion (red blood cells [RBCs] or whole blood), which is associated with a range of challenges that include ensuring safety of the blood supply (e.g., to avoid transfusion‐transmitted diseases), limited availability of suitable blood, inconvenience to both patients and healthcare professionals, and associated costs 3. Erythropoiesis‐stimulating agents (ESAs) such as darbepoetin alfa (DA) are among treatments that can increase serum hemoglobin (Hb) concentrations and thereby reduce the need for blood transfusions 3, providing benefit to patients and healthcare systems.

In 2008, the US Food and Drug Administration (US FDA) and the European Medicines Agency (EMA) added a boxed warning to the labels for ESAs, stating that ESAs may increase the risk of death, myocardial infarction, stroke, venous thromboembolism (VTE), thrombosis of vascular access, and tumor progression or recurrence 4, 5. At the same time, the US FDA updated DA's prescribing information, decreasing the Hb treatment initiation threshold to <10 g/dL and also added limitations of use, stating that DA was not indicated for patients receiving myelosuppressive chemotherapy when the anticipated outcome is cure 4. The EMA made similar changes to DA's summary of product characteristics, decreasing the Hb treatment initiation and discontinuation thresholds to ≤10 g/dL and >12 g/dL, respectively 5.

Since the updates to the DA labels to add the boxed warning and change Hb initiation and discontinuation thresholds, no clinical trials have been conducted to evaluate DA's efficacy when administered per the current labels. As an example, the key registration phase 3 placebo‐controlled trials of DA had included patients when their Hb was ≤11.0 g/dL, and the DA dose was withheld if a patient's Hb increased to >15.0 g/dL for men or >14.0 g/dL for women 6, 7. In these registration studies, DA administration was reinstated at 50% of the previous dose when the patient's Hb fell to ≤13.0 g/dL.

Several meta‐analyses and systematic reviews have assessed DA's safety and efficacy profile in the CIA setting 8, 9, 10, 11, 12, and most have reported that DA is safe and effective in this setting, and leads to improvements in Hb levels and/or reductions in the need for blood transfusions. The recent meta‐analysis by Pirker et al. 12 evaluated patient‐level data from four studies in patients with disease burden varying from Stage II or lower/limited to Stage III or higher/extensive and who had initiated DA at Hb ≤10 g/dL per EMA's summary of product characteristics for DA 5. However, no studies have evaluated the efficacy of DA when initiated at Hb ≤10 g/dL in patients with advanced disease and CIA, to meet the limitations of use for DA in the US; specifically that DA is not for use in patients with cancer receiving myelosuppressive chemotherapy when the anticipated outcome is cure 4.

In this retrospective subgroup analysis of clinical trial data, we examined the efficacy and safety of DA when administered at baseline Hb ≤10 g/dL in patients with Stage IV cancer.

## Methods

### Patients and study design

A review of a central database of Amgen‐sponsored DA trials in CIA described in Pirker et al. 12 had identified three Amgen‐sponsored randomized, placebo‐controlled, double‐blind, phase 3 clinical trials 7, 13, 14 that had included patients with Stage IV cancers and CIA who had initiated DA at Hb ≤10 g/dL. The three original studies had been approved by the relevant institutional review boards, and all patients who had participated in those studies had provided written, informed consent. Table 1 summarizes the key characteristics of the three original studies. Hernandez et al. 13 (Study 20030232) had enrolled patients with nonmyeloid malignancies receiving multicycle chemotherapy (DA [300 *μ*g, every 3 weeks], *n *= 193; placebo, *n *= 193). Vansteenkiste et al. 7 (Study 980297) had enrolled patients with lung cancer undergoing platinum‐containing chemotherapy (DA [2.25 *μ*g/kg, every week], *n *= 156; placebo, *n *= 158). Pirker et al. 14 (Study 20010145) had enrolled patients with extensive‐stage small‐cell lung cancer (SCLC) receiving first‐line platinum‐containing chemotherapy (DA [300 *μ*g, every week], *n *= 299; placebo, *n *= 298). For the current analysis, data for patients with Stage IV cancer who had been initiated on DA therapy or placebo at baseline Hb ≤10 g/dL were extracted from the original studies and pooled. Data from patients with hematologic cancers were excluded, and then data from the remaining subgroup of patients were analyzed.

**Table 1:** Key characteristics of the three randomized, controlled studies included in the analysis

Amgen study #/Reference	Tumor type	DA treatment duration (weeks)	Baseline Hb concentration for study inclusion (g/dL)	Initial DA dose and schedule	Hb indication for transfusion (g/dL)	Total enrolled patients who received study drug (*n*)		Patients with Hb ≤10 g/dL at baseline (*n*)		Percentage of patients with ≤10 g/dL at baseline (%)
						Placebo	DA	Placebo	DA	
20030232Hernandez et al. 13	Nonmyeloid malignancies^a^	15	<11	300 *μ*g, Q3W	≤8^b^	193	193	83	85	44
980297Vansteenkiste et al. 7	Lung cancer	12	≤11	2.25 *μ*g/kg, QW	≤8^c^	158	156	67	57	39
20010145Pirker et al. 14	Small‐cell lung cancer	18	≥9 and ≤13	300 *μ*g, QW	NS	298	299	18	12	5

DA, darbepoetin alfa; Hb, hemoglobin; NS, not specified; QW, every week; Q3W, every 3 weeks.

a Includes non‐small‐cell lung, small‐cell lung, ovarian, cervical, uterine/endometrial, breast, large intestine/colon, prostate, and pancreatic cancer and non‐Hodgkin's lymphoma and multiple myeloma.

b Transfusions at Hb >8 g/dL were allowed if signs or symptoms of anemia were present.

c Recommended Hb concentration for transfusion; initiation of transfusion was at the investigator's discretion.

### Study outcomes

The proportions of patients with an Hb increase of ≥1 g/dL and ≥2 g/dL and time to first Hb increase of ≥1 g/dL and ≥2 g/dL were assessed from initiation of treatment through 12 weeks. The proportions of patients receiving blood transfusions, either RBCs or whole blood, and time to first transfusion were assessed from the start of week 5 (day 29) through to week 12. Efficacy outcomes were also assessed by subgroups of patients who initiated DA treatment at Hb ≥9 to ≤10 g/dL versus those who initiated DA at Hb <9 g/dL. Safety was assessed by occurrence of adverse events (AEs).

### Statistical analysis

Crude and Kaplan–Meier (K‐M) proportions of patients who experienced each outcome were determined and 95% confidence intervals (CIs) were calculated; Wilson's Exact method was used for the crude CIs 15, and Greenwood's formula 16 of taking the 1 minus the survivor function at the last noncensored time point was used for time‐to‐event data. For the meta‐analysis, hazard ratios (HRs) from the fixed‐effects inverse‐variance model were used to compare results for patients who received DA versus patients who received placebo, and heterogeneity between studies was reported using the *I* ^2^ statistic.

## Results

### Patients

A total of 213 patients met the eligibility criteria for data extraction and analysis for this study: 100 from the Hernandez et al. study 13 that had enrolled patients with nonmyeloid malignancies (DA, *n *= 57; placebo, *n *= 43); 83 from the Vansteenkiste et al. study 7 that had enrolled patients with lung cancer (DA, *n *= 46; placebo, *n *= 37), and 30 from the Pirker et al. study 14 that had enrolled patients with extensive‐stage SCLC (DA, *n *= 12; placebo, *n* = 18). Table 2 summarizes baseline demographics and clinical characteristics of the identified patients. In total, 115 patients had received DA and 98 had received placebo. Of these, 157 had baseline Hb ≥9 to ≤10 g/dL (DA, *n *= 82; placebo, *n *= 75) and 56 had baseline Hb <9 g/dL (DA, *n* = 33; placebo, *n *= 23).

**Table 2:** Baseline demographics and clinical characteristics

Characteristic	Hb ≤10 g/dL(All patients)		Hb ≥9 to ≤10 g/dL		Hb <9 g/dL	
	Placebo*N *= 98	DA*N *= 115	Placebo*N *= 75	DA*N *= 82	Placebo*N *= 23	DA*N *= 33
Sex, *n* (%)						
Male	57 (58.2)	67 (58.3)	42 (56.0)	46 (56.1)	15 (65.2)	21 (63.6)
Female	41 (41.8)	48 (41.7)	33 (44.0)	36 (43.9)	8 (34.8)	12 (36.4)
Age, mean (SD), years	63.1 (10.4)	61.7 (11.0)	63.5 (11.0)	61.5 (11.8)	61.6 (8.5)	61.9 (8.8)
Age <65 years, *n* (%)	47 (48.0)	68 (59.1)	35 (46.7)	48 (58.5)	12 (52.2)	20 (60.6)
Race, white, *n* (%)	92 (93.9)	103 (89.6)	71 (94.7)	71 (86.6)	21 (91.3)	32 (97.0)
Study, *n* (%)						
Hernandez et al. 13	43 (43.9	57 (49.6)	35 (46.7)	42 (51.2)	8 (34.8)	15 (45.5)
Vansteenkiste et al. 7	37 (37.8)	46 (40.0)	23 (30.7)	29 (35.4)	14 (60.9)	17 (51.5)
Pirker et al. 14	18 (18.4)	12 (10.4)	17 (22.7)	11 (13.4)	1 (4.3)	1 (3.0)
Tumor type, *n* (%)						
Lung	63 (64.3)	64 (55.7)	46 (61.3)	46 (56.1)	17 (73.9)	18 (54.5)
Breast	9 (9.2)	22 (19.1)	9 (12.0)	16 (19.5)	0	6 (18.2)
Gastrointestinal	10 (10.2)	8 (7.0)	10 (13.3)	7 (8.5)	0	1 (3.0)
Genitourinary	4 (4.1)	5 (4.3)	2 (2.7)	2 (2.4)	2 (8.7)	3 (9.1)
Gynecologic	2 (2.0)	5 (4.3)	1 (1.3)	3 (3.7)	1 (4.3)	2 (6.1)
Other	10 (10.2)	11 (9.6)	7 (9.3)	8 (9.8)	3 (13.0)	3 (9.1)
ECOG performance status, *n* (%)						
0	9 (9.2)	14 (12.2)	8 (10.7)	12 (14.6)	1 (4.3)	2 (6.1)
1	50 (51.0)	54 (47.0)	42 (56.0)	39 (47.6)	8 (34.8)	15 (45.5)
2	24 (24.5)	20 (17.4)	15 (20.0)	12 (14.6)	9 (39.1)	8 (24.2)
3	0	2 (1.7)	0	1 (1.2)	0	1 (3.0)
Unknown	15 (15.3)	25 (21.7)	10 (13.3)	18 (22.0)	5 (21.7)	7 (21.2)
Baseline Hb, mean (SD), g/dL	9.2 (0.7)	9.2 (0.7)	9.6 (0.3)	9.6 (0.3)	8.2 (0.6)	8.4 (0.5)

DA, darbepoetin alfa; Hb, hemoglobin; SD, standard deviation; ECOG, Eastern Cooperative Oncology Group.

Over half of the patients in both groups were men (DA, 58.3%; placebo, 58.2%). Mean (SD) age was similar in the DA and placebo subgroups (61.7 [11.0] years and 63.1 [10.4] years, respectively, for all patients), but more patients in the DA versus the placebo subgroups were <65 years of age (59.1% vs. 48.0% for all patients; 58.5% vs. 46.7% in the baseline Hb ≥9 to ≤10 g/dL subgroup; and 60.6% vs. 52.2% in the baseline Hb <9 g/dL subgroup). Lung cancer was the most common malignancy (DA, 55.7%; placebo, 64.3% for all patients). More patients in the DA versus the placebo subgroup had breast cancer (DA, 19.1%; placebo, 9.2% for all patients). Baseline disease stage and Hb levels were similar for the DA and placebo subgroups. Overall, most patients had Eastern Cooperative Oncology Group (ECOG) performance status 0–1 versus ≥2 (59.6% [127 patients] vs. 21.6% [46 patients]), with unknown ECOG performance status for 40 (18.8%) of the patients.

### Hb response

**Figure 1:**
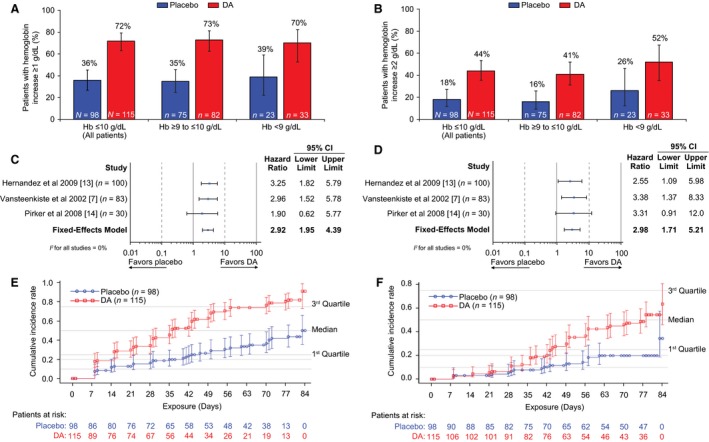
Hb response during treatment weeks 1 to 12.^a^ Crude proportions of patients with Hb increase of ≥1 g/dL (A) and ≥2 g/dL (B) were determined. Error bars are 95% CIs calculated based on Wilson's Exact method. Hazard ratios for Hb increase of ≥1 g/dL (C) and ≥2 g/dL (D) were determined from the fixed‐effects inverse‐variance model, with heterogeneity between studies reported using the *I* ^2^ statistic. K‐M plots of time to Hb increase of ≥1 g/dL (E) and ≥2 g/dL (F) are shown. ^a^Hb measurements within 28 days after a blood transfusion were excluded from the analysis. CI, confidence interval; DA, darbepoetin alfa; Hb, hemoglobin; K‐M, Kaplan–Meier.

In the first 12 treatment weeks, more patients in the DA subgroup versus the placebo subgroup achieved Hb increases of ≥1 g/dL and ≥2 g/dL for all patients (Fig. 1A and B, Table 3). Similar results were observed when data were analyzed by the subgroups of baseline Hb of ≥9 to ≤10 g/dL or <9 g/dL (Fig. 1A and B, Table 3). Overall, fixed‐effects model HRs for DA versus placebo for an Hb increase of ≥1 g/dL and ≥2 g/dL during the first 12 treatment weeks were 2.92, 95% CI: 1.95, 4.39 and 2.98, 95% CI: 1.71, 5.21, respectively (Fig. 1C and D). By study, Hb increases of ≥1 g/dL and ≥2 g/dL were significantly different for DA versus placebo in the subsets of patients from the Hernandez et al. study 13 (nonmyeloid malignancy; *n *= 100) and the Vansteenkiste et al. study 7 (lung cancer; *n *= 83) (Fig. 1C and D). However, this difference did not reach statistical significance in the subset of patients from the Pirker et al. study 14 (SCLC; *n *= 30) (Fig. 1C and D), probably due to the small population size.

**Table 3:** Crude and K‐M proportions of patients with Hb increase of ≥1 g/dL and ≥2 g/dL during treatment weeks 1 to 12.^a^

Outcome	Hb ≤ 10 g/dL(All patients)		Hb ≥9 to ≤10 g/dL		Hb <9 g/dL	
	Placebo*N *= 98	DA*N *= 115	Placebo*N *= 75	DA*N *= 82	Placebo*N *= 23	DA*N *= 33
Patients with Hb increase of ≥1 g/dL, *n*	35	83	26	60	9	23
Crude % (95% CI)^b^	36 (27, 46)	72 (63, 80)	35 (25, 46)	73 (63, 82)	39 (22, 59)	70 (53, 83)
K‐M % (95% CI)	50 (34, 65)	91 (78, 104)	49 (30, 68)	91 (77, 104)	59 (31, 87)	82 (68, 97)
Patients with Hb increase of ≥2 g/dL, *n*	18	51	12	34	6	17
Crude % (95% CI)^b^	18 (12, 27)	44 (36, 54)	16 (9, 26)	41 (31, 52)	26 (13, 47)	52 (35, 68)
K‐M % (95% CI)	34 (15, 54)	64 (45, 82)	34 (11, 58)	63 (40, 86)	35 (12, 59)	63 (44, 82)

a Hb measurements within 28 days after a blood transfusion were excluded from the analysis.

b 95% CIs were calculated based on Wilson's Exact method.

CI, confidence interval; DA, darbepoetin alfa; Hb, hemoglobin; K‐M, Kaplan–Meier.

Median time to Hb increase of ≥1 g/dL was shorter with DA (36 days) versus placebo (not evaluable, since <50% of patients achieved Hb increase of ≥1 g/dL) in all patients (Fig. 1E, Table 4). Similar results were observed for the subgroup of patients who initiated DA at baseline Hb ≥9 to ≤10 g/dL or <9 g/dL (Table 4). Similarly, median time to Hb increase of ≥2 g/dL was shorter for DA versus placebo (78 days vs. not evaluable) (Fig. 1F, Table 4).

**Table 4:** Median time to Hb increase of ≥1 g/dL and ≥2 g/dL.^a^

Outcome	Hb ≤10 g/dL(All patients)		Hb ≥9 to ≤10 g/dL		Hb <9 g/dL	
	Placebo*N *= 98	DA*N *= 115	Placebo*N *= 75	DA*N *= 82	Placebo*N *= 23	DA*N *= 33
Median time (95% CI) to Hb increase of ≥1 g/dL, days	NE (NE, NE)	36 (29, 43)	NE (NE, NE)	36 (23, 43)	72 (55, NE)	43 (29, 50)
Median time (95% CI) to Hb increase of ≥2 g/dL, days	NE (NE, NE)	78 (57, NE)	NE (NE, NE)	78 (57, NE)	NE (NE, NE)	50 (43, NE)

CI, confidence interval; DA, darbepoetin alfa; Hb, hemoglobin; NE, not evaluable.

a Hb measurements within 28 days after a blood transfusion were excluded from the analysis.

### Blood transfusions

**Figure 2:**
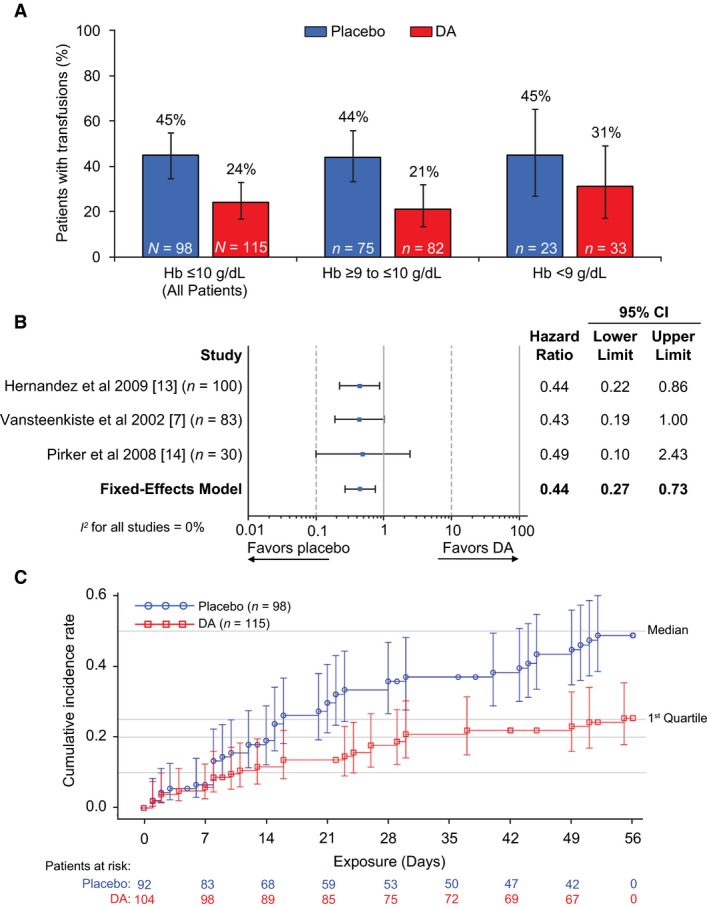
RBC or whole blood transfusions between week 5 and week 12.^a^ (A) Crude proportions of patients who received RBC or whole blood transfusions were determined. Error bars are 95% CIs calculated based on Wilson's Exact method. (B) Hazard ratios for receiving RBC or whole blood transfusions were determined from the fixed‐effects inverse‐variance model, with heterogeneity between studies reported using the *I* ^2^ statistic. (C) K‐M plot of time to receiving first RBC or whole blood transfusion is shown. ^a^Hb measurements within 28 days after a blood transfusion were excluded from the analysis. CI, confidence interval; DA, darbepoetin alfa; Hb, hemoglobin; K‐M, Kaplan–Meier.

The proportion of patients who received RBC or whole blood transfusions between week 5 and week 12 was lower in the DA subgroup compared with the placebo subgroup, regardless of baseline Hb at which DA was initiated (≤10 g/dL [all patients], ≥9 to ≤10 g/dL, or <9 g/dL) (Fig. 2A, Table 5). Fixed‐effects model HR for DA versus placebo for receiving RBC or whole blood transfusions between week 5 and week 12 was 0.44, 95% CI: 0.27, 0.73 (Fig. 2B). The K‐M plot of time to receiving first RBC or whole blood transfusion between week 5 and week 12 is shown in Figure 2C. A lower proportion of patients in the DA subgroup than in the placebo subgroup received RBC or whole blood transfusions with time. Median time to receiving first transfusion was not evaluable as <50% of patients in each subgroup received transfusions (DA, 24%; placebo, 45%; Fig. 2A).

**Table 5:** Crude and K‐M proportions of patients who received RBC or whole blood transfusions between week 5 and week 12.^a^

	Hb ≤10 g/dL (All patients)		Hb ≥9 to ≤10 g/dL		Hb <9 g/dL	
	Placebo*N *= 98	DA*N *= 115	Placebo*N *= 75	DA*N *= 82	Placebo*N *= 23	DA*N *= 33
Patients who received blood transfusions, *n*	41	25	31	16	10	9
Crude % (95% CI)^b^	45 (35, 55)	24 (17, 33)	44 (33, 56)	21 (14, 32)	45 (27, 65)	31 (17, 49)
K‐M % (95% CI)	49 (38, 60)	25 (17, 34)	48 (35, 60)	23 (13, 33)	53 (30, 75)	32 (15, 50)

a Hb measurements within 28 days after a blood transfusion were excluded from the analysis.

b 95% CIs were calculated based on Wilson's Exact method.

CI, confidence interval; DA, darbepoetin alfa; Hb, hemoglobin; K‐M, Kaplan–Meier; RBC, red blood cell.

### Safety

Table 6 shows AEs occurring in >10% of all patients combined. The most common AEs in all patients were nausea (70 patients [32.9%]), vomiting (57 patients [26.8%]), dyspnea (47 patients [22.1%]), and fatigue (46 patients [21.6%]). Most AEs occurred at similar frequencies between the DA and placebo subgroups or were slightly lower in the DA subgroup versus the placebo subgroup: nausea (34 patients [29.8%] vs. 36 patients [36.4%]), vomiting (28 patients [24.6%] vs. 29 patients [29.3%]), dyspnea (22 patients [19.3%] vs. 25 patients [25.3%]), constipation (15 patients [13.2%] vs. 21 patients [21.2%]), anemia (13 patients [11.4%] vs. 19 patients [19.2%]), and neutropenia (13 patients [11.4%] vs. 15 patients [15.2%]). AEs that occurred at a slightly higher frequency in the DA subgroup versus the placebo subgroup include back pain (16 patients [14.0%] vs. 9 patients [9.1%]), peripheral edema (16 patients [14.0%] vs. 11 patients [11.1%]), and pyrexia (15 patients [13.2%] vs. 10 patients [10.1%]).

**Table 6:** Adverse events occurring in >10% of all patients

Adverse event	Placebo*N *= 99^a^ *n* (%)	DA*N *= 114^a^ *n* (%)	All patients*N *= 213*n* (%)
Nausea	36 (36.4)	34 (29.8)	70 (32.9)
Vomiting	29 (29.3)	28 (24.6)	57 (26.8)
Dyspnea	25 (25.3)	22 (19.3)	47 (22.1)
Fatigue	23 (23.2)	23 (20.2)	46 (21.6)
Constipation	21 (21.2)	15 (13.2)	36 (16.9)
Anemia	19 (19.2)	13 (11.4)	32 (15.0)
Decreased appetite	17 (17.2)	18 (15.8)	35 (16.4)
Diarrhea	15 (15.2)	19 (16.7)	34 (16.0)
Asthenia	15 (15.2)	17 (14.9)	32 (15.0)
Neutropenia	15 (15.2)	13 (11.4)	28 (13.1)
Peripheral edema	11 (11.1)	16 (14.0)	27 (12.7)
Pyrexia	10 (10.1)	15 (13.2)	25 (11.7)
Insomnia	10 (10.1)	12 (10.5)	22 (10.3)
Back pain	9 (9.1)	16 (14.0)	25 (11.7)

DA, darbepoetin alfa.

a One patient randomized to DA did not receive treatment, and was included with the placebo group for safety analyses.

For the AEs of interest, deep vein thrombosis occurred in 1 (0.9%) patient with DA versus 2 (2.0%) patients with placebo, arterial thrombosis occurred in 1 (0.9%) patient with DA versus none with placebo, venous thrombosis occurred in none of the patients with DA and 1 (1.0%) patient with placebo, and thrombosis occurred in 1 (0.9%) patient with DA and none with placebo.

## Discussion

To the best of our knowledge, our study is the first to evaluate the efficacy and safety of DA when treatment is initiated at baseline Hb ≤10 g/dL in patients with Stage IV cancer and CIA. Results from our study confirm that DA is more effective than placebo at increasing Hb levels and at reducing the need for RBC or whole blood transfusions in patients with Stage IV cancer and CIA when DA treatment is initiated at baseline Hb ≤10 g/dL. Similar results were observed when patients were evaluated by subgroups of those who initiated DA at baseline Hb ≥9 to ≤10 g/dL versus <9 g/dL.

In this study, analysis of efficacy outcomes was limited to data up to 12 weeks as the DA treatment duration in one of the studies, Vansteenkiste et al. 7, was 12 weeks, with DA treatment of up to 15 weeks and 18 weeks in the studies in Hernandez et al. 13 and Pirker et al. 14, respectively (Table 1). As demonstrated in earlier studies, DA treatment for up to 12 weeks can result in achievement of efficacy outcomes including Hb increases and reductions in the need for blood transfusions 6, 7, 17, 18, 19. RBC or whole blood transfusion needs were assessed starting from week 5 (day 29) to allow adequate time for DA‐stimulated erythropoiesis and production of sufficient RBCs to avoid the need for transfusion 17, 18, 19.

In this study, we evaluated data from subsets of patients with Stage IV cancer who had participated in the studies reported in Hernandez et al. 13, Vansteenkiste et al. 7, and Pirker et al. 14. A recent meta‐analysis by Pirker et al. 12 evaluated data from patients who had participated in the same three studies 7, 13, 14 and an additional fourth study by Hedenus et al. 6, focusing on a more heterogeneous patient population that included patients with early‐stage to advanced disease. Consistent with the findings from this study, Pirker et al. 12 reported that more patients treated with DA versus placebo achieved Hb increases of ≥1 g/dL (HR: 2.07, 95% CI: 1.62, 2.63) and ≥2 g/dL (HR: 2.91, 95% CI: 2.09, 4.06), and that less patients treated with DA versus placebo received blood transfusions (HR: 0.58, 95% CI: 0.44, 0.77). Also, that study demonstrated similar efficacy outcomes regardless of whether DA was initiated at baseline Hb 9 to ≤10 g/dL or <9 g/dL. Similar findings were reported from a post hoc analysis of a phase 3 study in patients with nonmyeloid malignancies and CIA who initiated DA treatment at baseline Hb <10 g/dL, with K‐M transfusion incidences of 36% for DA administered every 3 weeks and 41% for DA administered every week 20. These observations demonstrate DA's effectiveness in improving Hb levels and decreasing transfusion needs in patients with both early‐stage as well as advanced disease. Data from our study are also consistent with data reported in studies of DA in patients where treatment was initiated at a broader range of baseline Hb, including those in which DA treatment was initiated at baseline Hb >10 g/dL 7, 13, 21, 22, 23.

Overall, no new safety issues associated with DA use were identified in this study, with most AEs occurring with similar frequencies in the DA and placebo subgroups or at higher frequencies in the placebo subgroup versus the DA subgroup. AEs that occurred at a slightly higher frequency in the DA subgroup versus the placebo subgroup include back pain, peripheral edema, and pyrexia.

Of note, deep vein thrombosis occurred in two patients in the placebo subgroup and one patient in the DA subgroup, and venous thrombosis occurred in one patient with placebo and in none of the patients with DA. Thrombosis occurred in one patient and arterial thrombosis occurred in one patient in the DA subgroup, with no patients experiencing these events in the placebo subgroup. In general, patients with advanced cancer have a 20% risk of VTE during the course of their disease 24, 25. As such, the 3% VTE rate (6 of 213 patients) observed in this study that evaluated patients with Stage IV cancer is surprisingly low. Reasons for the low VTE rate are not apparent, but contributing factors may include baseline age (54.0% of patients were <65 years vs. 46.0% who were ≥65 years of age), ECOG performance status (59.6% of patients had ECOG performance status of 0–1 vs. 21.6% with ECOG performance status of ≥2) (Table 2), and limited follow‐up period (patients were followed during the short study period only, which ranged from 12 weeks to 18 weeks).

There are limitations to note with regards to this analysis. First, the studies that met eligibility criteria for analysis were three distinct Amgen‐sponsored DA trials for which patient‐level data were available in‐house. Secondly, these three studies had been designed to evaluate DA initiation at baseline Hb levels mainly >10 g/dL and in patients at all disease stages. As such, small numbers of patients from each study met the eligibility criteria for data extraction in our analysis; that is, patients with Stage IV cancers and CIA and who initiated DA at Hb ≤10 g/dL. Thirdly, the three studies had enrolled patients with different tumor types (100 with nonmyeloid malignancies 13, 83 with lung cancer 7, and 30 with extensive‐stage SCLC 14) and had administered DA at different doses and schedules (300 *μ*g, every 3 weeks 13; 2.25 *μ*g/kg, every week 7; and 300 *μ*g, every week 14) (Table 1). Lastly, outcomes such as survival and quality of life were not analyzed in this study. However, the main advantage of this analysis is that pooling of data from the three studies included here allowed an evaluation of the efficacy and safety of DA when administered at baseline Hb ≤10 g/dL in patients with Stage IV cancer, in the absence of trial data addressing this important clinical question.

In conclusion, results of this study confirm the efficacy and safety of DA when administered in patients with cancer who are receiving chemotherapy only if Hb is ≤10 g/dL. Patients receiving DA had appropriate Hb responses and reductions in the incidence of RBC or whole blood transfusions. No new safety findings with DA use were reported even though DA was initiated at low baseline Hb levels (≤10 g/dL) in the subgroup of patients with stage IV cancer evaluated in this study.

## Conflict of interest

Chet Bohac is an employee and stockholder of Amgen Inc. Laura Belton is a contractor funded by Amgen (Europe) GmbH. Ralph V. Boccia reports receiving research funding from Amgen Inc., Celgene, AstraZeneca, Onyx, TG Therapeutics, and Genentech and was on the Speaker's Bureau for Amgen Inc. and Onyx. David H. Henry reports participating in an advisory board for Amgen Inc. Hassan H. Ghazal has no conflicts of interest to declare.
